# Three-Dimensional Printed Hybrid Scaffolds with Layered Polycaprolactone/Nanosized Smectic Clay Nanocomposite and Chitosan/Collagen/Demineralized Bone Powder Hydrogels Targeting Osteochondral Tissue Engineering

**DOI:** 10.3390/jfb16120441

**Published:** 2025-11-26

**Authors:** Thiago Ferreira Cândido Lima Verde, Matheus Ribeiro Viana, André Sales Aguiar Furtado, Guilherme de Castro Brito, Manuel Henrique de Sousa Cunha, Livia Alves Filgueiras, Anderson Nogueira Mendes, Fernanda Roberta Marciano, Caio Moreira de Souza, Thiago Domingues Stocco, Anderson Oliveira Lobo

**Affiliations:** 1 Interdisciplinary Laboratory for Advanced Materials, Materials Science & Engineering Graduate Program, Federal University of Piauí, Teresina 64049-550, Brazil; thiagovalantysor@gmail.com (T.F.C.L.V.); matheusrviana@ufpi.edu.br (M.R.V.); andre.furtado@ifma.edu.br (A.S.A.F.); guilhermme.brito@hotmail.com (G.d.C.B.); henriquemanuel975@gmail.com (M.H.d.S.C.); 2 Laboratory of Innovation in Science and Technology, Department of Biophysics and Physiology, Federal University of Piauí, Teresina 64049-550, Brazil; livia.filgueirass@gmail.com (L.A.F.); anderson.mendes@ufpi.edu.br (A.N.M.); 3 Department of Physics, Federal University of Piauí, Teresina 64049-550, Brazil; marciano@ufpi.edu.br; 4 Bioengineering Program, Scientific and Technological Institute, Brasil University, São Paulo 08230-030, Brazil; caiomoreira30@gmail.com

**Keywords:** tissue engineering, nanocomposites, 3d printing, hydrogels, articular cartilage, bone

## Abstract

This study addresses the challenges of osteochondral tissue engineering by developing a hybrid scaffold with intercalated layers of poly(ε-caprolactone) (PCL) in combination with different concentrations of nanosized synthetic smectic clay (Lap) and a hydrogel of chitosan, collagen and demineralized bone powder (DBP). The scaffold design specifically targets the critical junction between subchondral bone and calcified cartilage and utilizes the mechanical strength of PCL/Lap nanocomposites and the bioactivity of the chitosan/collagen/DBP hydrogel to support tissue regeneration. The PCL/Lap nanocomposite, characterized by increased hydrophilicity, improved swelling behavior, and enhanced stiffness, provides a robust scaffold, while the hydrogel layers improve bioactivity and fluid retention. Three-dimensional printing technology was used to fabricate multi-layer scaffold, ensuring interfacial cohesion between the layers. Rheological, morphological, chemical, and mechanical characterizations confirmed the successful integration of the materials and the mechanical suitability for the subchondral environment. Biocompatibility assays demonstrated the non-hemolytic nature of the scaffolds and a favorable trend in cell viability with increasing Lap content. This study presents a novel scaffold design that effectively combines mechanical stability and biological functionality. It fulfills the complex requirements of osteochondral repair and offers a promising platform for future tissue engineering strategies.

## 1. Introduction

Tissue engineering has proven to be a promising approach to meet the urgent need for effective treatments in regenerative medicine, with applications in various tissues such as cartilage and bone [1,2]. These tissues play an important role in maintaining the structural integrity and function of the musculoskeletal system. However, while bone has a remarkable capacity for self-repair, particularly in the case of minor injuries, its regenerative potential is significantly limited in the case of large-scale or complex defects [2]. Articular cartilage, on the other hand, has only a very limited ability to repair itself after injury or degeneration, which makes its regeneration a particularly urgent problem in tissue engineering [3].

The creation of scaffolds that can mimic both the mechanical and biological properties of native tissue is essential for the advancement of tissue engineering strategies. Such scaffolds must provide adequate mechanical support to withstand physiological stresses while creating a favorable environment for cell attachment, proliferation and differentiation [4,5]. This challenge is particularly pronounced in osteochondral defects, where both the articular cartilage and the underlying subchondral bone are affected and require simultaneous regeneration. Conventional scaffold materials are often inadequate as they either lack sufficient mechanical strength or do not support the necessary biological activity [6,7]. This dual challenge requires the development of innovative materials and scaffold designs that effectively bridge the gap between mechanical robustness and bioactivity.

Synthetic polymers such as poly(ε-caprolactone) (PCL) are frequently used due to their mechanical robustness, ease of processing and adjustable degradation rate. PCL is particularly valued for its high tensile strength and ductility, making it suitable for applications where long-term structural support is required. However, PCL is inherently hydrophobic, which can limit its interaction with cells and biological tissue, posing a challenge for effective tissue integration [8,9]. This limitation necessitates strategies to improve the hydrophilicity and bioactivity of PCL-based scaffolds. To overcome these challenges, natural polymers such as chitosan and collagen are often incorporated into scaffold constructs. These naturally derived materials exhibit excellent biocompatibility, biodegradability and inherent bioactivity, making them ideal candidates for improving the biological performance of synthetic scaffolds [10,11]. Chitosan, a chitin derivative, is known for its antimicrobial properties and its ability to promote wound healing, while collagen, the primary structural protein of the extracellular matrix, is a natural substrate for cell attachment and growth [10,12,13]. The combination of synthetic and natural polymers aims to create scaffolds that are not only mechanically stable but also enhance biological response, improving tissue integration and regeneration.

In recent years, the incorporation of nanomaterials such as Lap, a synthetic layered silicate, has gained attention due to its ability to further improve the properties of polymer-based scaffolds. Lap can increase the hydrophilicity of hydrophobic polymers such as PCL, thereby improving cell affinity and promoting more effective tissue integration. In addition, the unique nanoscale structure of Lap can facilitate the uniform dispersion of nanoparticles in the polymer matrix, which can improve the mechanical properties of the scaffold without compromising its bioactivity. This dual improvement, mechanical and biological, makes Lap a valuable additive in the development of advanced scaffolds for tissue engineering applications [14,15,16]. Another important component in the development of bioactive scaffolds is demineralized bone powder (DBP). DBP is obtained from bone tissue from which the mineral content has been removed. What remains is a matrix rich in collagen and growth factors, which are crucial for bone regeneration. The incorporation of DBP into scaffolds improves their osteoconductive properties and promotes the adhesion, proliferation and differentiation of bone cells [17,18]. By integrating DBP with biopolymers such as chitosan and collagen, the scaffold not only mimics the natural bone environment, but also supports the regeneration of bone tissue by providing the necessary biochemical cues. This makes DBP a key material for scaffolds developed for bone tissue engineering, particularly in applications that require both structural support and biological functionality [19,20].

Hybrid scaffolds which combine materials with different properties offer a promising strategy for tissue engineering. By combining mechanically robust materials such as PCL with bioactive hydrogels, these scaffolds are designed to provide both structural support and a favorable environment for tissue regeneration [21,22]. However, the development of such scaffolds poses a major challenge, particularly when hydrophobic materials such as PCL are combined with hydrophilic hydrogels. The layering of these materials can be particularly advantageous. This approach is increasingly being explored, with 3D printing playing a crucial role in the production of multi-material, multi-layered scaffolds. The precision and versatility of 3D printing allows for careful placement of the individual layers of material, ensuring strong adhesion and cohesion without compromising the overall structural integrity of the scaffold [23,24,25,26,27]. This method is particularly promising for applications where both mechanical strength and biological activity need to be integrated, particularly for heterogeneous tissues, such as osteochondral tissue engineering.

The current study thus builds on these advances by developing a hybrid scaffold with intercalated layers of PCL/Lap and chitosan/collagen/DBP. This design takes advantage of the complementary properties of these materials: PCL/Lap provides the mechanical strength necessary to support physiological loads, while the chitosan/collagen/DBP hydrogel increases bioactivity and hydrophilicity, creating a more favorable environment for cell attachment and proliferation. In addition, the scaffold integrates recognized key effector components for bone tissue engineering, including bioactive ions released from nanosized smectic clay (e.g., magnesium and silicate), which stimulate osteogenic signaling; natural growth factors from DBP, particularly bone morphogenetic proteins (BMPs), which promote osteoblastic differentiation; cell adhesion motifs (e.g., RGD sequences) present in collagen, which support cellular attachment and proliferation; and chitosan, which, although not a signaling molecule per se, contributes through its amine groups by adsorbing serum glycoproteins that modulate cell–material interactions [10,13,17,28].

By combining these materials in a layered scaffold, we aim to create a structure that not only fulfills the mechanical requirements of bone and cartilage, but also promotes tissue integration and regeneration. This approach specifically targets the critical transition between subchondral bone and calcified cartilage, which is a key challenge in osteochondral defects. By trying to mimic the different properties of these two layers, our design attempts to overcome the dual challenge of providing both mechanical stability and biological functionality, thus providing a novel solution to the complex problems of osteochondral repair. The aim of this study is to fabricate and characterize these hybrid scaffolds, evaluate their potential for tissue engineering applications and contribute to the development of more effective strategies for osteochondral regeneration.

## 2. Materials and Methods

### 2.1. Preparation of PCL/Lap Nanocomposite

PCL with a molecular weight of 80 kDa (Sigma-Aldrich, St. Louis, MO, USA) was used as the polymer matrix. The PCL pellets were dissolved in chloroform (Synth, Diadema, SP, Brazil) at an initial concentration of 10% (*w*/*v*) PCL with magnetic stirring at room temperature. The dissolution was carried out until a homogeneous and clear solution was obtained, ensuring complete dissolution of the polymer. Separately, Laponite^®^ XLG (BYK Additives, Wesel, NRW, Germany), a synthetic phyllosilicate, was dispersed in 3 mL of chloroform. Dispersion was achieved by vigorous magnetic stirring to prevent agglomeration of the Lap nanoparticles, following the methodology used in previous studies [29,30,31,32,33].

Once both components were prepared, the Lap dispersion was gradually added to the PCL solution with constant stirring. This step was crucial to ensure an even distribution of Lap in the polymer matrix. The resulting mixture was magnetically stirred for a further period of time to improve the interaction between PCL and Lap particles. After the mixing process, the solvent (chloroform) was evaporated under controlled conditions in a fume hood to prevent contamination and ensure safety.

Three different concentrations of Lap were tested in this study: 0.0% (pure PCL), 0.5%, 1.0% and 1.5% (*w*/*v*), referred to as PCL, PCL/Lap 0.5, PCL/Lap 1.0 and PCL/Lap 1.5, respectively. These low concentrations were selected based on previous studies that demonstrated enhanced mechanical and biological performance of polymer–Lap nanocomposites at similar levels [29,34,35,36]. The prepared PCL/Lap nanocomposites were then stored in airtight containers to prevent moisture absorption and degradation prior to use.

### 2.2. Formulation of Chitosan/Collagen/DBP Hydrogel

The formulation of the chitosan/collagen hydrogel and its variant with DBP involved a series of precise steps to ensure homogeneity and optimal material properties. Chitosan powder (Polymar, Fortaleza, CE, Brazil) was first dissolved in an acetic acid solution (Sigma-Aldrich, St. Louis, MO, USA) at a concentration of 2% (*w*/*v*) with constant magnetic stirring at room temperature. The dissolution process was carried out for one hour to ensure complete solubilization of the chitosan, resulting in a clear and viscous solution. After preparing the chitosan solution, collagen type I (Sigma-Aldrich, St. Louis, MO, USA) was added to the system at a concentration of 1% (*w*/*v*). The collagen was gradually added to the chitosan solution with constant stirring for 30 min.

To formulate the chitosan/collagen/DBP hydrogel, DBP was first prepared according to the method of Lee et al. [20]. In brief, a bovine thigh bone was obtained from a local butcher, and all residual tissue and fat were carefully removed with a scalpel. The cleaned bone was then scalded in a saline solution containing sodium chloride (NaCl) for two hours to further purify the bone matrix. It was then washed in deionized water for a further hour. The bone was then dried in an oven at 100 °C. It was then mechanically chopped with a serrated saw to obtain fine bone particles. These particles were then sieved through an analytical sieve with a mesh size of 400 to obtain a uniform particle size distribution. The bone powder was then mixed in a 1:1 solution of acetone (Sigma-Aldrich, St. Louis, MO, USA) and Milli-Q water with vigorous stirring for two hours. The demineralized bone powder was then filtered using filter paper and a funnel and then dried and freeze-dried to obtain a fine, dry powder.

This DBP was then incorporated into the previously prepared chitosan/collagen hydrogel at a concentration of 0.5% (*w*/*v*). The DBP was added to the hydrogel stepwise for 30 min under constant magnetic stirring to ensure uniform distribution in the matrix.

### 2.3. Fabrication of Hybrid 3D Printed Scaffolds

The scaffold was designed using SolidWorks Standard version software and sliced with Slic3r software version 1.0.1. This resulted in a cylindrical structure with a diameter of 7 mm, a total height of 1.6 mm and a rectilinear fill pattern with a printed layer height of 0.2 mm. The hybrid scaffolds were produced using a 3D bioprinter equipped with an extrusion-based printing system (Allevi 2, Allevi, Philadelphia, PA, USA). Two independent print heads were used for the 3D printing process, each configured to dispense different materials.

The first print head was intended for the application of the PCL/Lap nanocomposite, which was filled into a metal syringe and dispensed through a 23G metal needle. The PCL/Lap nanocomposite was heated to a controlled temperature range of 80–90 °C and held for 15–20 min prior to application to ensure good flowability and extrusion consistency. Once the PCL/Lap layer was applied, the framework had to cool and solidify completely before the hydrogel layer could be applied.

The second print head was used to apply the chitosan/collagen/DBP hydrogel. This material was filled into a plastic syringe and applied at room temperature through a conical 25G plastic needle. After the chitosan/collagen/DBP hydrogel layer was printed, the hydrogel was cross-linked using the freeze–thaw technique [37]. The scaffold was placed in a freezer at −20 °C and completely frozen for 2 h. After this time, the scaffold was removed and thawed for a further 2 h at room temperature.

**Figure 1:**
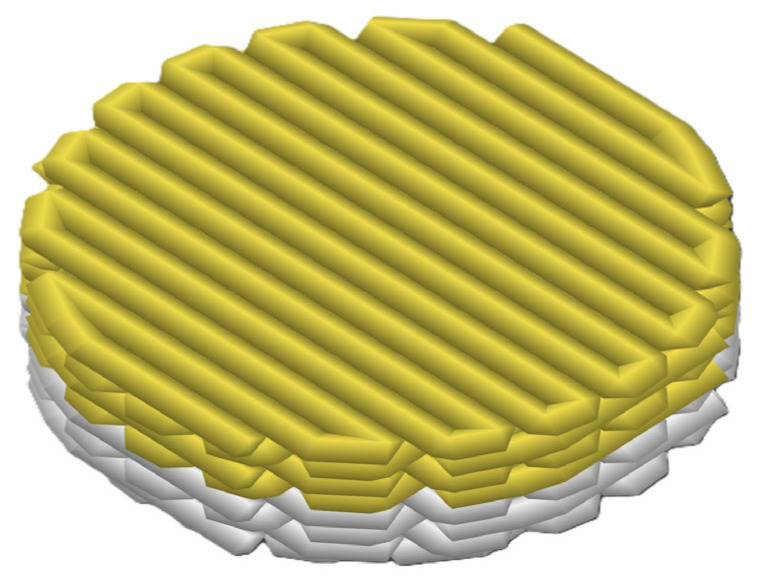
Schematic representation of the structure of the hybrid scaffold, showing the layer-by-layer approach. The lower layers represent the printed PCL/Lap nanocomposite (white layer), while the upper layers represent the printed chitosan/collagen/DBP hydrogel (yellow layer).

In this layer-by-layer approach, four consecutive layers of PCL/Lap nanocomposite were printed first, which together formed the bottom layer of the scaffold, followed by the printing of four more layers of chitosan/collagen/DBP hydrogel, which formed the top layer (Figure 1).

For the comparative analysis, scaffolds were produced from PCL/Lap nanocomposites with three different Lap concentrations (0.5%, 1.0% and 1.5%) and with and without hydrogel layers. A qualitative visual inspection confirmed that the overall scaffold dimensions closely match the CAD design (Figure 1). Minor variances in filament deposition reflect nozzle diameter tolerances and cooling-induced shrinkage, without affecting the scaffold’s macroscopic geometry.

### 2.4. Analyzes and Characterizations

The fabricated hybrid 3D-printed scaffolds were subjected to characterization and analysis as described in the following sections.

#### 2.4.1. Rheological Analysis

The rheological measurements of the solutions were carried out with a rheometer (AR-G2, TA Instruments, New Castle, DE, USA) equipped with a parallel plate geometry with a gap setting of 0.3 mm. The viscosity of the samples was measured over a range of 0.1 to 8 s^−1^ to evaluate the shear thinning behavior of the formulations. Each formulation was carefully loaded onto the bottom plate of the rheometer to ensure that as little air as possible was entrapped. The PCL/lap samples were measured at a controlled temperature of 80–90 °C to replicate the conditions during 3D extrusion, while the hydrogel samples were measured at room temperature. All samples were kept in equilibrium for 5 min prior to measurement to ensure thermal and mechanical stability.

#### 2.4.2. Contact Angle Measurement

The wettability of the scaffolds was evaluated by measuring the angle of contact with water at intervals of 0 and 5 s. The analysis was performed to compare the surface hydrophilicity of PCL/Lap nanocomposites with and without Laps. The images of the water droplets on the scaffold surfaces were taken with a macro camera under cold light illumination to minimize any thermal effects on the droplet shape. The images were then processed and analyzed using ImageJ version 1.53t software, whereby the contact angles were precisely measured.

#### 2.4.3. Morphological Analysis by Scanning Electron Microscopy (SEM)

The morphological properties of the scaffold were examined using a scanning electron microscope (SEM) (FEI Quanta FEG 250, FEI Company, Hillsboro, OR, USA) at an acceleration voltage of 20 kV. Specimens were attached to aluminum stubs with double-sided carbon tape to ensure stable positioning during imaging. Qualitative assessment of microstructural features was based on images obtained from at least three independent experiments.

#### 2.4.4. Fourier-Transform Infrared Spectroscopy (FTIR)

The chemical composition and functional groups of the PCL/Lap nanocomposites with different Lap concentrations and the final hybrid scaffolds including the chitosan/collagen/DBP hydrogel layers were analyzed by Fourier transform infrared spectroscopy (FTIR) in attenuated total reflectance (FTIR-ATR) mode with a Bruker Vertex 70 spectrometer (Bruker, Billerica, MA, USA). The spectra were recorded over a wavenumber range of 500–4000 cm^−1^ and averaged over 60 scans to ensure a high signal-to-noise ratio. The spectral data was processed and analyzed using ORIGINPro 9 software (OriginLab Corporation, Northampton, MA, USA).

#### 2.4.5. Swelling Test

The swelling behavior of the scaffolds were evaluated by immersing the specimens in phosphate-buffered saline (PBS, pH 7.4) at a controlled temperature of 37 °C. Samples were weighed at regular intervals of 1, 2, 3, 4, 5, 6 and 7 h to monitor water uptake over time. Prior to immersion, the dry weight of each sample was recorded. After each time interval, samples were gently blotted to remove excess surface liquid and then weighed immediately. The degree of swelling was calculated as the percentage increase in weight relative to the original dry weight. This test was performed in triplicate to ensure reproducibility and accuracy of results.

#### 2.4.6. Mechanical Testing

Mechanical compression tests were performed to evaluate the effect of Lap concentration and the presence of chitosan/collagen/DBP hydrogel layers on the stiffness of scaffolds. The tests were conducted using a universal testing machine (EMIC, São José dos Pinhais, PR, Brazil) equipped with a 200 kN load cell. Cylindrical specimens (15 mm high × 7 mm in diameter) were printed by interleaving several layers (Supplementary Video S1). The layers followed the same manufacturing parameters described in Section 2.1 and Section 2.3; several layers were printed until the minimum recommended desired height for use in the universal machine was reached. The main mechanical parameter evaluated was Young’s modulus, calculated from the slope of the linear region of the stress–strain curve obtained during the test. Young’s modulus (determined in this study) is equivalent to the modulus of elasticity, according to ASTM D695 [38] and ISO 604 [39] standards.

#### 2.4.7. Biocompatibility Assessment: Hemolysis Test

The hemolysis test was conducted following the protocols outlined by Mendes et al. [40] and Batista et al. [41]. In brief, scaffold samples were incubated in 2.0 mL tubes containing 0.9% NaCl saline and 100 μL of erythrocytes from mammalian arterial blood (Canis lupus familiaris). The use of erythrocytes was approved by the Ethics Committee for Research of the Federal College of Piauí (Protocol No. 463/18). The samples were incubated at 37 °C for 1 h to simulate physiological conditions. After incubation, the tubes were centrifuged at 3000 rpm for 5 min, and the supernatant was carefully transferred to a 96-well plate. Hemoglobin release in the supernatant was quantified using a GloMax Microplate Reader (Promega, Madison, WI, USA) at a wavelength of 560 nm. Saline served as a negative control (0% hemolysis), while a 1% detergent solution was used as a positive control (100% hemolysis). This assay provided a quantitative measure of the hemolytic potential of the scaffolds, which is crucial for evaluating their biocompatibility in tissue engineering applications.

#### 2.4.8. In Vitro Cytocompatibility Assessment: MTT Assay

The in vitro cytocompatibility of the final hybrid scaffold was evaluated using the 3-(4,5-dimethylthiazol-2-yl)-2,5-diphenyltetrazolium bromide (MTT) assay, in accordance with ISO 10993-5:2009 [42] and ISO 10993-12:2021 [43] standards for biological evaluation of medical devices. All procedures were conducted in compliance with the ethical principles established by the Brazilian National Council for the Control of Animal Experimentation (CONCEA), at the Integrated Center for Morphology and Stem Cell Research (NUPCELT) of the Federal University of Piauí (UFPI).

Pre-osteoblastic MC3T3-E1 cells (subclone 4) were purchased from the Cell Bank of Rio de Janeiro (BCRJ, Brazil) and expanded in α-MEM (Minimum Essential Medium Eagle—Alpha modification, Sigma-Aldrich, St. Louis, MO, USA) supplemented with 10% fetal bovine serum (FBS, Sigma-Aldrich, St. Louis, MO, USA) and 100 U/mL penicillin–streptomycin (LGC Biotecnologia, Cotia, SP, Brazil), under standard incubation conditions (37 °C, 5% CO_2_). Upon reaching approximately 80% confluence, cells were harvested and seeded at a density of 10,000 cells/well into 96-well plates, where sterilized hybrid scaffolds were already positioned. UV sterilization of scaffolds was performed for 30 min on each side prior to use.

Negative controls consisted of cells cultured in the absence of scaffolds, while blank wells containing only medium served as background reference. Cell viability was assessed after 24, 48, and 72 h of incubation. At each time point, 0.5 mg/mL of MTT solution (Sigma-Aldrich, St. Louis, MO, USA) was added to each well and incubated for 4 h. Subsequently, formazan crystals formed by metabolically active cells were solubilized with 500 μL of dimethyl sulfoxide (DMSO, Sigma-Aldrich, St. Louis, MO, USA) and incubated for 10 min under agitation. Aliquots of 100 μL were transferred to new 96-well plates, and absorbance was read at 595–600 nm using a microplate spectrophotometer (BioTek ELx800, Winooski, VT, USA). Cell viability was calculated relative to the negative control and expressed as a percentage.

### 2.5. Statistical Analysis

All experiments were performed in triplicate and results are expressed as mean ± standard deviation (SD). Statistical analysis was performed with GraphPad Prism 7 software (GraphPad Software Inc., Boston, MA, USA) using two-way ANOVA. A *p*-value of less than 0.05 was considered statistically significant. This procedure was applied uniformly to all data sets.

## 3. Results and Discussion

The development of PCL/Lap nanocomposites and chitosan/collagen/DBP hydrogels was the basis for the fabrication of the hybrid 3D-printed scaffolds investigated in this study. In the fabrication of PCL/Lap nanocomposites, PCL was dissolved in chloroform and then Lap nanoparticles were dispersed in the polymer matrix. This process ensured a homogeneous distribution of Lap, which is crucial for improving the mechanical properties and printability of the resulting scaffolds. In parallel, chitosan/collagen/DBP hydrogels were formulated by first dissolving chitosan in acetic acid and then incorporating type I collagen and demineralized bone powder (DBP). The aim of this methodical preparation was to obtain a hydrogel with optimal viscoelastic properties and bioactivity suitable for integration into the scaffold structure.

**Figure 2:**
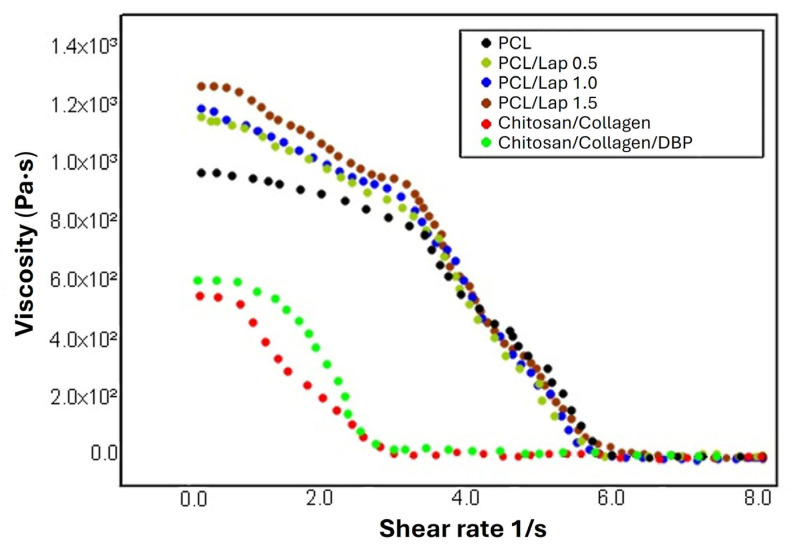
Rheological analysis as a function of shear rate for PCL/Lap nanocomposites with different Lap concentrations (0.5%, 1.0% and 1.5%) and chitosan/collagen hydrogels with and without DBP.

The rheological characterization of solutions is a crucial technique to assess the mechanical properties of materials in response to applied stress or strain. In this study, a rheological analysis was performed to evaluate the viscoelastic properties of the PCL/Lap nanocomposite and chitosan/collagen hydrogels both with and without DBP. The results presented in Figure 2 show the rheological behavior of the PCL solution and its nanocomposites with different Lap concentrations (0.5%, 1.0% and 1.5%) as well as the chitosan/collagen hydrogel and the chitosan/collagen/DBP hydrogel.

As can be seen in Figure 2, all solutions exhibited viscoelastic behavior, although the viscosity and elasticity of the different formulations varied. The PCL solution exhibited a base viscosity which progressively increased with increasing concentration of Lap. This increase in viscosity can be attributed to the higher degree of dispersion of the nanoparticles in the polymer matrix, which leads to increased interactions between the polymer chains and the Lap platelets. The shear-thinning behavior observed in the PCL/Lap nanocomposites, as reported in other studies [44,45], points to its potential for extrusion-based 3D printing, where the material must flow easily under shear stress but remain structurally stable after extrusion [46,47,48]. In this context, rheological behavior is not only indicative of structural properties but also critical for printability. For extrusion-based systems, printable formulations typically exhibit viscosities in the range of 0.03 to >60,000 Pa·s, depending on material type and shear conditions [49,50]. More specifically, for effective material deposition during extrusion, viscosity should decrease under shear, ideally falling within the range of 0.3 to 30 Pa·s, as higher values may require excessive extrusion pressure and lower values may impair filament formation and stability [51,52]. When at rest or under very low shear rates, ideal formulations typically present viscosities between 100 and 10,000 Pa·s, providing sufficient mechanical integrity and shape retention after deposition [53]. In our results, all formulations fall within these established rheological windows, presenting higher viscosity at rest and a consistent decrease across the shear rate range tested, confirming their suitability for extrusion-based fabrication and structural stability post-printing. In the case of the chitosan/collagen hydrogels, the addition of DBP led to a slight increase in viscosity at low shear rates, while the viscosity profiles converged at higher shear rates. This subtle increase may be attributed to interactions between DBP particles and the chitosan/collagen matrix, possibly through hydrogen bonding or other intermolecular forces, contributing to improved structural integrity and shape retention after extrusion [54]. Thus, this characteristic is beneficial for the application of the hydrogel in tissue engineering, as it supports dimensional stability in the post-printing phase. Rheological analysis thus provides important insights into the mechanical properties and processability of these materials, particularly in the context of 3D printing and tissue engineering applications.

**Figure 3:**
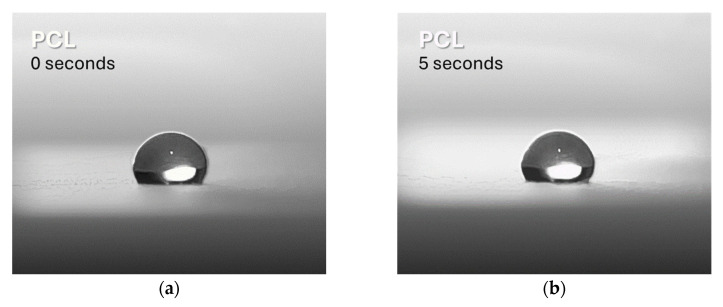

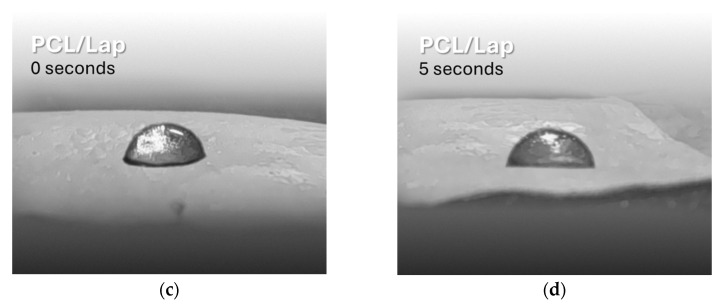
Representative contact angle measurements showing the changes in wettability of pure PCL (**a**,**b**) and PCL/Lap 0.5 nanocomposite (**c**,**d**) at 0 and 5 s, respectively. Statistical significance was found between the pure PCL and PCL/Lap 0.5 nanocomposite groups (*p* < 0.05), with no significant differences observed between the different Lap concentrations.

Contact angle measurement is used to evaluate the wettability of a surface, which is an important indicator of its hydrophilicity or hydrophobicity. In this study, this technique was used to investigate the change in hydrophilicity of PCL after the addition of Lap. The results indicate that the surface of pure PCL is hydrophobic, as shown by the higher measured contact angle (120° ± 3° at 0 s and 121° ± 2° at 5 s, Figure 3a,b, respectively), which is confirmed by previous studies [55,56]. After the addition of Lap, a significant decrease in the contact angle was observed, reflecting an increase in the hydrophilicity of the surface (82° ± 2° at 0 s and 86° ± 3° at 5 s, Figure 3c and 3d, respectively). This result was expected as Lap is inherently more hydrophilic than PCL [14]. The hydrophilicity of the surface is crucial for the interactions between cells and structures, particularly for cell adhesion, which is closely related to the surface energy of the material [57,58,59]. Furthermore, improving the hydrophilicity of the PCL/Lap nanocomposite is crucial for its subsequent integration with the hydrogel layers in the scaffold, as a hydrophobic surface could hinder this interaction [60]. No significant differences were found between the different Lap concentrations, so only representative images of PCL and PCL/Lap 0.5 are shown in Figure 3.

**Figure 4:**
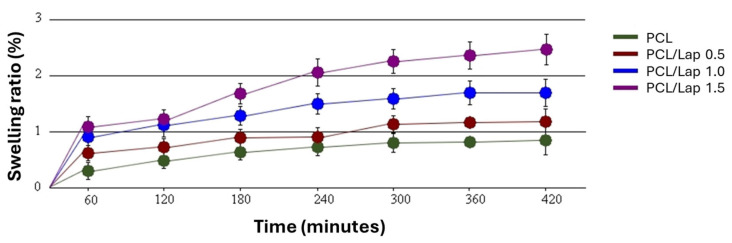
Swelling behavior of PCL/Lap nanocomposites with different Lap concentrations (0.0%, 0.5%, 1.0% and 1.5%) over a period of 7 h. A statistically significant increase in swelling was observed with higher Lap content, particularly after 7 h (*p* < 0.05).

Building on the findings from the contact angle measurements, where the addition of Lap significantly increased the surface hydrophilicity of the PCL nanocomposites, the swelling behavior further supports this observation. As can be seen in Figure 4, the swelling ability of the PCL/Lap nanocomposites in PBS increased with the Lap concentration over a 7 h period, reflecting the greater affinity for water absorption due to the increased hydrophilicity caused by Lap. This trend emphasizes the hydrophilic nature of Lap, which facilitates water uptake within the nanocomposite matrix, as shown by the significant decrease in contact angle (Figure 3c,d). This improved swelling behavior is particularly important for applications where controlled hydration and expansion of the scaffold material is desired [61].

**Figure 5:**
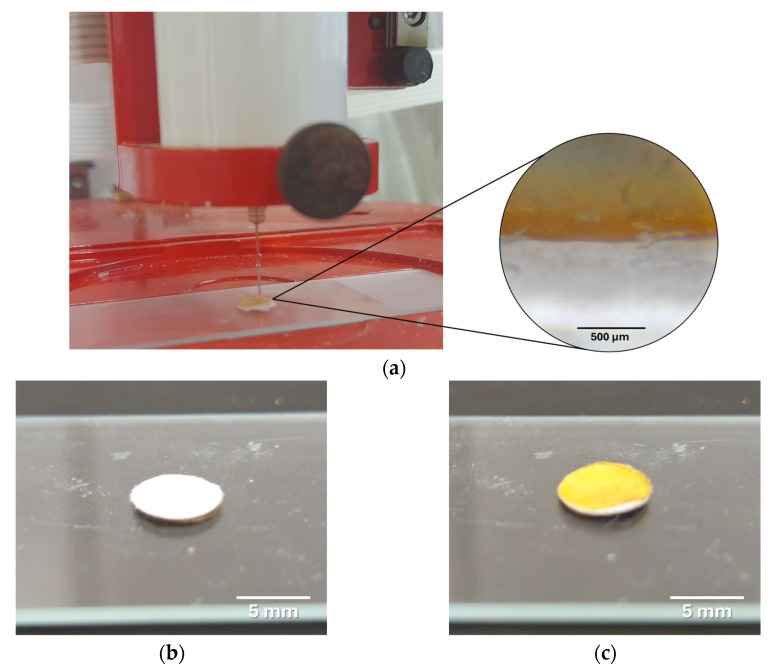
Comprehensive illustration of the 3D-printed hybrid scaffold. (**a**) Shown is the 3D printing process with a magnified section taken with an optical microscope, illustrating the interface between the PCL/Lap nanocomposite layer and the chitosan/collagen/DBP hydrogel layer, highlighting the integration between the two materials. (**b**) Photo of the printed scaffold consisting only of the PCL/Lap nanocomposite and (**c**) the corresponding scaffold with the chitosan/collagen/DBP hydrogel layer deposited on top.

The fabrication of hybrid 3D-printed scaffolds using the extrusion-based method successfully resulted in structures with alternating layers of PCL/Lap nanocomposite and chitosan/collagen/DBP hydrogel. The layer-by-layer approach proved to be a reliable and reproducible method for fabricating complex scaffolds with visible integration between the different material layers, as shown in Figure 5. This integration is crucial for ensuring the overall structural integrity of the scaffold for calcified cartilage and bone tissue, as it promotes cohesion between the mechanically robust PCL/Lap layers, which are hard like bone, and the bioactive hydrogel layers, which are soft like articular cartilage.

**Figure 6:**
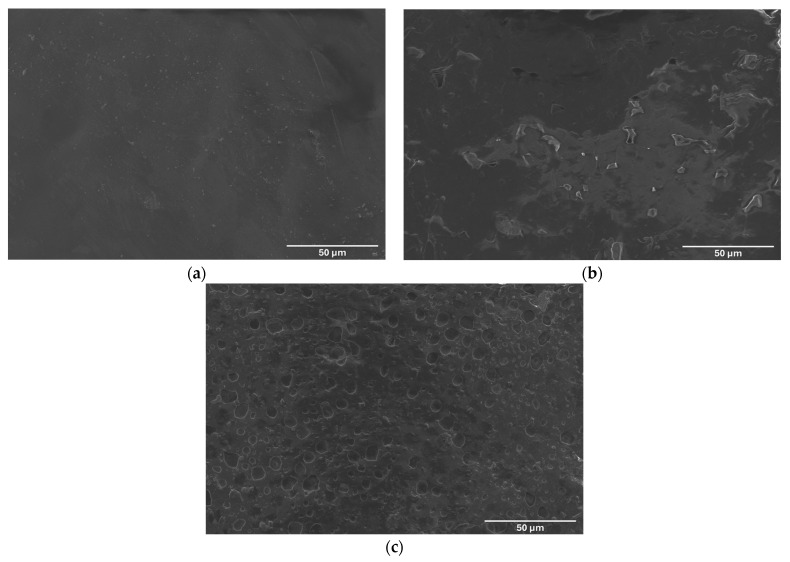
Scanning electron microscope (SEM) images showing the surface morphology of (**a**) pure PCL scaffold, (**b**) PCL/Lap nanocomposite scaffold and (**c**) the upper surface of the chitosan/collagen/DBP hydrogel layer in the hybrid scaffold composed of PCL/Lap nanocomposite and hydrogel.

The surface morphology of the scaffolds was analyzed by SEM to evaluate the structural differences resulting from the incorporation of Lap and the integration of chitosan/collagen/DBP hydrogel layers. Figure 6a shows the surface of the pure PCL scaffold, which has a relatively smooth and homogeneous texture characteristic of the amorphous nature of PCL. The lack of significant surface features indicates limited interaction with external substances, which is consistent with the hydrophobicity observed in the contact angle measurements. In contrast, Figure 6b shows the PCL/Lap nanocomposite scaffold, where the addition of Lap significantly altered the surface morphology. The presence of Lap particles can be seen as small, scattered features on the surface, contributing to a rougher texture. This increase in surface roughness is likely responsible for the increased hydrophilicity mentioned earlier, as the rough surface increases the surface area available for interaction with water molecules [62]. Figure 6c presents the surface morphology of the chitosan/collagen/DBP hydrogel layer at the top of the scaffold. The observed porous architecture is characteristic of crosslinked chitosan and collagen hydrogels, as confirmed by SEM in previous studies [63,64,65]. These pore-like structures are beneficial for tissue engineering applications as they can improve nutrient diffusion and waste removal and create a more favorable environment for cell attachment and proliferation [61,66]. The presence of these pores indicates that the scaffold supports improved tissue integration and vascularization, both of which are crucial for the successful regeneration of cartilage and bone tissue.

**Figure 7:**
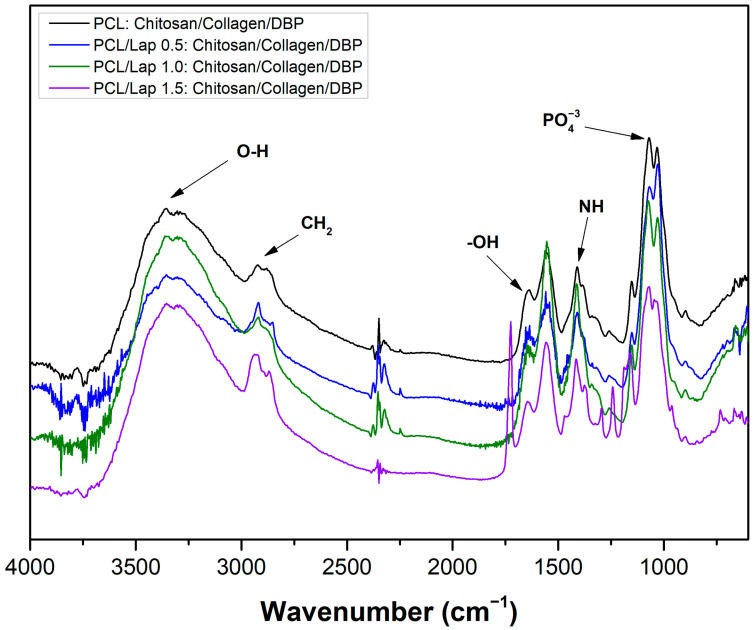
FTIR spectra of hybrid scaffolds: PCL: chitosan/collagen/DBP scaffold, PCL/Lap 0.5: chitosan/collagen/DBP, PCL/Lap 1.0: chitosan/collagen/DBP scaffold and PCL/Lap 1.5: chitosan/collagen/DBP.

FTIR was used to analyze the chemical interactions within the hybrid scaffolds of PCL, Lap, chitosan, collagen and DBP. The spectra obtained (Figure 7) showed distinct absorption bands corresponding to the different functional groups in the scaffold components, indicating successful integration of the materials. The broad bands between 3400 cm^−1^ and 3000 cm^−1^ are attributed to the O-H stretching vibrations, which are mainly associated with the hydroxyl groups in chitosan and collagen as well as the adsorbed water molecules in the Lap layers [15,67,68,69]. The specific peak near 3360 cm^−1^, which is conspicuous in all samples, can be associated with the amide A band and O-H stretching, indicating the presence of hydrogen bonding within the hydrogel matrix. The asymmetric and symmetric CH_2_ stretching vibrations observed at 2920 cm^−1^ are characteristic of the PCL backbone and confirm the preservation of the chemical structure of the polymer after integration with Lap and the hydrogel layers [30,70]. In addition, the bands observed at approx. 1635 cm^−1^ correspond to the bending vibrations of the adsorbed water molecules (−OH), which indicate the hydrated structure of the Lap [15]. Further analysis of the spectra between 1600 cm^−1^ and 1400 cm^−1^ revealed bands associated with N-H bending vibrations, probably originating from the collagen component. These bands indicate the presence of amide II and III vibrations [68,69], which confirm the successful incorporation of collagen into the hydrogel layers. The presence of phosphate groups (PO_4_^3−^) identified by absorption bands near 1200 cm^−1^ indicates the integration of bone-derived components from the DBP, which are essential for promoting osteoconductivity in bone tissue engineering [71,72]. In the PCL/Lap 1.5: chitosan/collagen/DBP spectrum, a distinct absorption band was also observed in the range of 1730–1750 cm^−1^, which may be attributed to C=O stretching from residual acetic acid used in the preparation of the hydrogel layer. This band was not evident in other formulations, suggesting that minor solvent retention may have occurred at this concentration. However, as further detailed in the biological characterization section, this group demonstrated no signs of hemolysis and showed favorable cellular viability, suggesting that the presence of this band does not compromise scaffold performance. Additionally, the slight shifts in the peaks corresponding to the O-H, CH_2_ and PO_4_^3−^ bands in the Lap-containing scaffolds indicate physical interactions between the Lap nanoparticles and the surrounding polymer matrix [30]. These shifts may indicate enhanced hydrogen bonding and electrostatic interactions, which are critical for maintaining the structural integrity of the scaffold and the uniform distribution of bioactive components. Overall, the FTIR analysis confirms the successful production of hybrid scaffolds with well-integrated chemical components.

**Figure 8:**
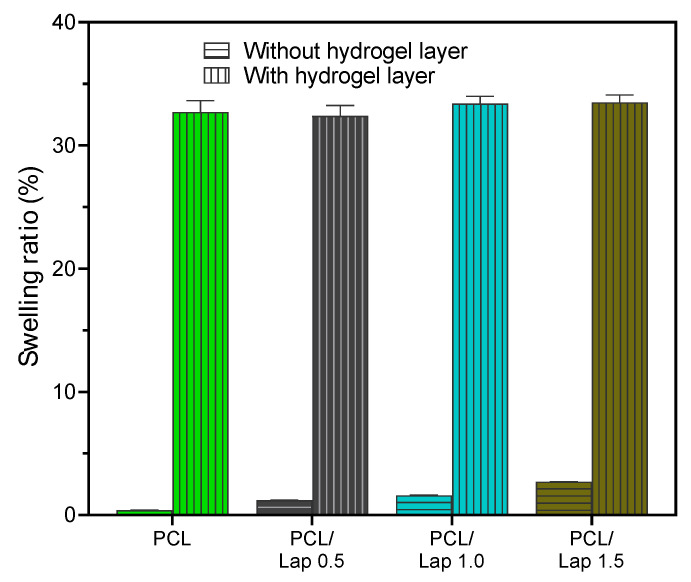
Comparative swelling behavior of hybrid scaffolds with and without hydrogel layers after 7 h in PBS. The presence of chitosan/collagen/DBP hydrogel layers significantly increased the swelling capacity in all groups, with statistically significant differences observed between the groups with hydrogel layers and those without hydrogel layers (*p* < 0.05). No significant differences were observed between the different concentrations of Lap within the hydrogel-containing groups.

Extending the previous analysis of the swelling behavior of the PCL/Lap nanocomposite, the incorporation of chitosan/collagen/DBP hydrogel layers significantly influenced the overall swelling ability of the hybrid scaffolds. As can be seen in Figure 8, the percentage of swelling after 7 h in PBS increased dramatically for the scaffolds with intercalated hydrogel layers compared to the scaffolds without hydrogel layers, with all hydrogel-containing groups exhibiting a statistically significant higher swelling capacity regardless of the Lap concentration. This pronounced swelling behavior is consistent with the intrinsic properties of hydrogels, which are known for their high-water absorption capacity and their ability to store large amounts of water in their network structure [61]. The significant increase in swelling observed in the hybrid scaffolds indicates that the hydrogel layers dominate the overall swelling response and effectively enhance the ability of the scaffold to absorb and bind liquids. This property is particularly beneficial for cartilage tissue engineering applications where a hydrated environment is fundamental to mimic the natural extracellular matrix and support cell viability and proliferation. In addition, the scaffold’s ability to swell and maintain hydration could facilitate the diffusion of nutrients and bioactive molecules throughout the construct, further promoting tissue regeneration and integration [61,73]. The lack of significant differences between the different concentrations of Lap in the hydrogel-containing frameworks underlines the predominant role of hydrogel in determining the swelling behavior. This observation suggests that although Lap contributes to the hydrophilicity of the nanocomposite, the hydrogel layers exert a much stronger influence on the overall swelling dynamics of the scaffold.

**Figure 9:**
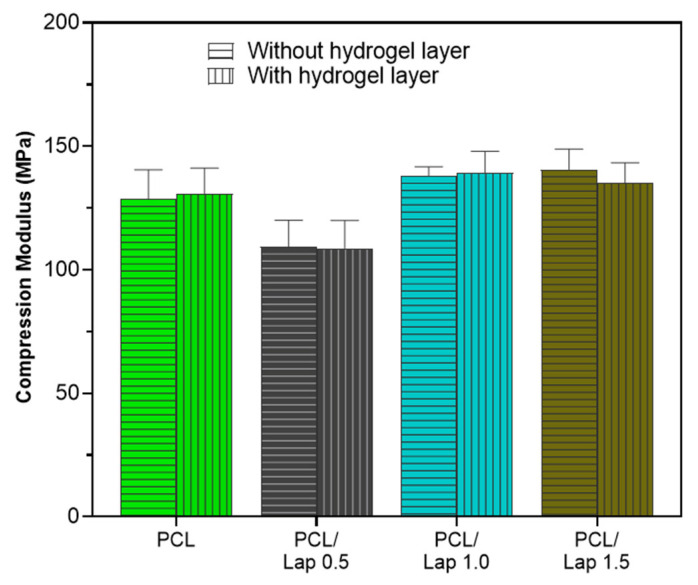
Young’s modulus of scaffolds composed of pure PCL and PCL reinforced with increasing concentrations of Lap (0.5%, 1.0%, and 1.5%), with and without the addition of chitosan/collagen/DBP hydrogel layers.

The mechanical performance of the scaffolds was evaluated through uniaxial compression tests, with the aim of investigating the influence of laponite (Lap) concentration on the structural stiffness of the printed samples. As shown in Figure 9, the Young’s modulus under compression varied according to the Lap content incorporated into the PCL matrix, highlighting the reinforcement potential conferred by argyle to the composite.

The pure PCL scaffolds presented a compression modulus of 130.8 ± 13.6 MPa, values consistent with those reported in the literature for PCL structures obtained by 3D printing, in which the mechanical performance strongly depends on the printing parameters, infill density and scaffold architecture. With the incorporation of 0.5%, 1.0%, and 1.5% (*w*/*v*) of Lap, the elastic moduli obtained were 108.7 ± 13.6 MPa, 139.1 ± 13.6 MPa, and 135.0 ± 13.6 MPa, respectively. The formulation containing 1.0% and 1.5% Lap showed the highest modulus and compressive strengths, confirming the reinforcing effect promoted by nanoclay when adequately dispersed in the polymer matrix.

The sample containing 0.5% Lap showed the lowest compressive modulus (108.7 MPa) among the formulations evaluated, suggesting that very low concentrations of the nanoreinforcement were not sufficient to generate a significant improvement in structural stiffness [74]. This behavior may be related to the partial dispersion of the nanoparticles and the low density of interfacial anchoring points between laponite and PCL, which reduces the efficiency of stress transfer between the phases. Furthermore, small variations in the thickness of the printed layers or in the uniformity of the filling may have contributed to this difference, especially considering the high sensitivity of the modulus to the microstructure of the scaffolds [74]. Thus, although a significant increase in modulus was observed at 1.0% Lap, it is not possible to determine whether the reinforcing effect begins precisely at this concentration, since intermediate values between 0.5% and 1.0% were not evaluated.

When comparing scaffolds with and without the hydrogel layer (chitosan/collagen/DBP), no measurable differences were observed in the compression modulus values; however, this comparison does not rely on statistical testing and should therefore be interpreted qualitatively. Although hydrogels are typically soft materials, the presence of the layer did not measurably reduce the overall stiffness of the samples, indicating that the mechanical behavior of the assembly is predominantly controlled by the PCL/Lap polymeric phase [75]. This result suggests that the interfacial adhesion between the hydrogel and the matrix was sufficient to maintain structural integrity under compressive load, but that the hydrogel, due to its viscoelastic nature, acts passively, without significantly contributing to the increase in the system’s stiffness [76]. Thus, the addition of hydrogel can be considered beneficial from a biological point of view, without compromising the mechanical performance of the scaffolds.

**Figure 10:**
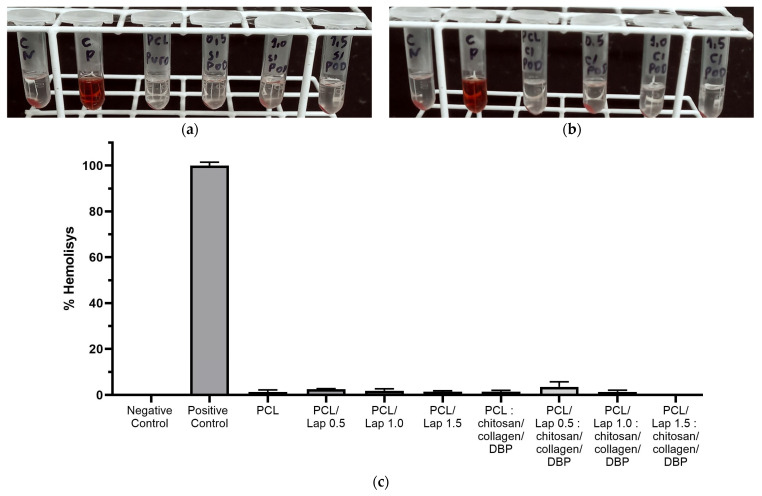
Evaluation of hemolysis of scaffold formulations: (**a**) Representative image of hemolysis test for PCL/Lap nanocomposites without hydrogel layers, including negative control, positive control, PCL, PCL/Lap 0.5, PCL/Lap 1.0 and PCL/Lap 1.5 groups; (**b**) Representative image of hemolysis test for PCL/Lap nanocomposites with chitosan/collagen/DBP hydrogel layers, in the same group order; (**c**) Quantitative hemolysis rates for all scaffold formulations. All formulations showed hemolysis rates below 5%, indicating good blood compatibility and classifying them as non-hemolytic. No statistically significant differences were found between the scaffold groups tested.

The hemolysis test provides crucial insights into the blood compatibility of biomaterials as it measures the potential of a material to cause the bursting of erythrocytes and the subsequent release of hemoglobin into the surrounding medium. This is a crucial parameter when assessing the safety of materials intended for biomedical applications, especially when they encounter blood [77,78]. According to the results shown in Figure 10, the hemolysis rates for all scaffold formulations, including those with different Lap concentrations and with or without chitosan/collagen/DBP hydrogel layers, were below 5%, which classifies them as non-hemolytic [79]. This low hemolytic activity indicates that the scaffold materials have good blood compatibility, which is crucial for their potential use in clinical applications. The base material, PCL, is known for its good hemocompatibility [80], and the addition of Lap did not affect this property. In addition, the addition of chitosan/collagen/DBP hydrogel layers did not significantly alter the hemolytic behavior of the scaffolds, further confirming their safety profile. In these areas, the scaffolds may encounter blood during implantation or in highly vascularized tissue, such as bone. To avoid inflammatory reactions and support successful integration into the host tissue, it is crucial that the scaffold materials do not induce hemolysis [81]. The absence of significant hemolytic activity in all tested scaffold formulations underlines their low toxicity towards erythrocytes, suggesting that these materials have a low risk of inducing blood-related cytotoxicity, making them promising candidates for applications in the field of calcified cartilage and bone tissue.

**Figure 11:**
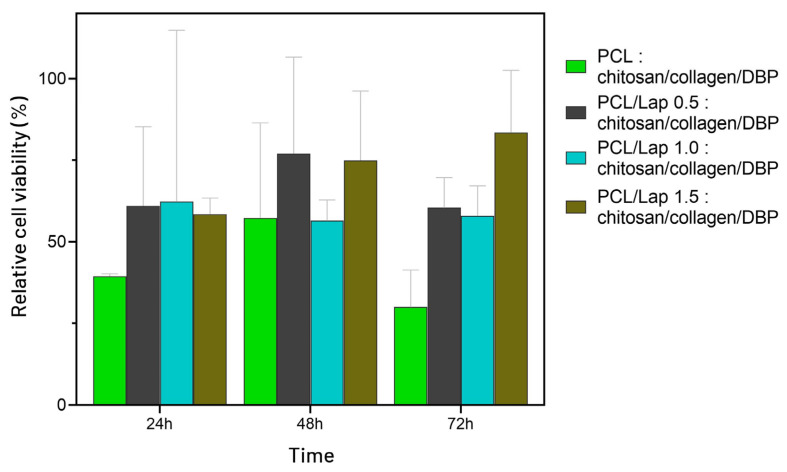
Cell viability of MC3T3-E1 pre-osteoblastic cells exposed to hybrid scaffolds composed of PCL and chitosan/collagen/DBP, with different concentrations of Lap (0.0%, 0.5%, 1.0%, and 1.5%), after 24, 48, and 72 h of incubation. Results are expressed as percentage of viability relative to the untreated control group. No statistically significant difference was found between the groups after applying one-way ANOVA (*p* > 0.05).

The MTT assay was used to assess the cytocompatibility of the hybrid scaffolds after 24, 48, and 72 h of incubation. As shown in Figure 11, the PCL–chitosan/collagen/DBP scaffold (without Lap) exhibited low cell viability values, ranging from 40% at 24 h to 30% at 72 h. Although these biopolymers are widely recognized for their intrinsic biocompatibility when used individually, their combination in this scaffold did not yield the expected cellular response. In contrast, the presence of Lap was associated with a tendency toward improved cell viability. Although no statistical differences were detected among groups, the biological trend aligns with previous studies reporting the beneficial effects of Lap on cell viability, likely due to enhanced ionic buffering, surface hydrophilicity, and favorable cell–material interactions [82,83,84]. The PCL/Lap 0.5 and 1.0 scaffolds exhibited intermediate behavior, with viability values between 56% and 78%, suggesting a transition toward cytocompatibility. The PCL/Lap 1.5 formulation demonstrated the most favorable result, maintaining a consistent cytocompatible profile with viability levels of 75% and 84% at 48 h and 72 h, respectively, both above the ISO 10993-5 [42] threshold of 70%, supporting its selection as the most promising candidate for use in osteochondral tissue engineering.

Among the tested formulations, the PCL/Lap 1.5: chitosan/collagen/DBP scaffold demonstrated the most balanced performance, combining the highest Young’s modulus, favorable swelling behavior, and hydrophilicity. Although rheological, hemolysis rates, and contact angle differences among the PCL/Lap groups were subtle, only the PCL/Lap 1.5: chitosan/collagen/DBP group exceeded the cytocompatibility threshold. Therefore, this concentration may be considered optimal within the tested range, offering the most favorable set of physical and biological properties for osteochondral scaffold development.

Although the results of this study show the successful fabrication and characterization of hybrid 3D-printed scaffolds, there were some challenges and limitations. One major technical challenge was that the PCL/Lap layer, which was printed at 80–90 °C, had to cool and solidify before the hydrogel layer could be applied. This step lengthened the overall printing time and presents an opportunity for optimization for future work, possibly by refining the cooling process or exploring alternative materials that may allow for faster transitions between layers. Also, although indirect evidence from rheological, and mechanical, assessments suggested homogeneous dispersion, we acknowledge that the absence of direct evaluation may represent a limitation of this study and, in future works, will incorporate high-resolution SEM and elemental mapping to directly validate the spatial distribution of Lap within the PCL matrix. Furthermore, the biological evaluation of the scaffolds was limited to the hemolysis test and MTT assay. However, future studies should focus on more specific in vitro tests relevant to cartilage and bone tissue, including cell adhesion, proliferation and differentiation, to evaluate the regenerative potential of the scaffold. In addition, in vivo studies will be crucial to validate the scaffold’s efficacy in promoting tissue regeneration and integration in a physiological environment. Furthermore, future studies could investigate strategies to modulate the spatiotemporal presentation of key effector molecules within the scaffold, such as gradient structuring, controlled release systems, and bioresponsive materials, aiming to regulate cell behavior throughout different stages of osteochondral tissue regeneration. Finally, exploring new formulations of hydrogels in future studies could further enhance the bioactivity of the scaffold and pave the way for more robust and versatile tissue engineering applications. Addressing these areas will be crucial to advancing the clinical relevance and applicability of these hybrid scaffolds.

## 4. Conclusions

The development and characterization of hybrid 3D-printed scaffolds composed of alternating layers of PCL/Lap nanocomposites and chitosan/collagen/DBP hydrogels represent a significant advance in the field of cartilage and bone tissue engineering. The successful fabrication of these scaffolds using a layer-by-layer extrusion-based printing process demonstrated the potential of combining mechanically robust and bioactive materials to create structures that closely mimic the complexity of native tissue. The integration of Lap into the PCL matrix not only improved the hydrophilicity and swelling capacity of the scaffolds but also enhanced their stiffness in a concentration-dependent manner, with Young’s modulus values reaching the range of native subchondral bone. Additionally, the incorporation of chitosan/collagen/DBP hydrogels improved scaffold microstructure and fluid retention, which are critical for creating a hydrated environment conducive to tissue regeneration. This is particularly important for osteochondral tissue engineering, where the scaffold must support the regeneration of both subchondral bone and calcified cartilage, two regions with different mechanical and biological requirements.

Furthermore, biocompatibility assessments demonstrated that the scaffolds were non-hemolytic and exhibited a trend toward improved cytocompatibility with increasing Lap content, with the PCL/Lap 1.5: chitosan/collagen/DBP scaffold surpassing the threshold for cell viability. These findings support the potential of this formulation as the most promising candidate for further preclinical investigations.

However, this study has also highlighted areas that need to be explored in the future. These include optimizing the printing process to reduce manufacturing time and the need for more extensive biological studies to fully determine the regenerative potential of these scaffolds. In summary, the hybrid scaffolds developed in this study represent a potential platform for cartilage and bone tissue engineering that can be flexibly adapted for a variety of regenerative applications. Future research should build on these results to further refine the scaffold design and validate its efficacy in vivo to ultimately advance the clinical translation of these innovative materials.

## Data Availability

The data presented in this study are available on request from the corresponding author.

## Supplementary Materials

The following supporting information can be downloaded at: https://www.mdpi.com/article/10.3390/jfb16120441/s1, Video S1: Mechanical tests.
